# Observational Study on Actual Cancer Screening Participation and Outcomes Among Patients with Lung Cancer Based on Linkage of Cancer Registry and Kyoto City Integrated Database Data from 2014 to 2018

**DOI:** 10.3390/ijerph22101595

**Published:** 2025-10-21

**Authors:** Tomonari Shimamoto, Yukiko Tateyama, Daisuke Kobayashi, Keiichi Yamamoto, Norihiro Nishioka, Yoshimitsu Takahashi, Hiroaki Ueshima, Kosuke Sasaki, Kosuke Kiyohara, Takeo Nakayama, Taku Iwami

**Affiliations:** 1 Department of Preventive Services, School of Public Health, Kyoto University, Kyoto 606-8501, Japan; shimamoto.tomonari.5w@kyoto-u.ac.jp (T.S.); nishioka.norihiro.26x@kyoto-u.jp (N.N.); 2 Department of Environmental Health and Global Health, Faculty of Pharmaceutical Sciences, Teikyo University, Tokyo 173-8605, Japan; 3 Medical Service Center, Ritsumeikan University, Kyoto 603-8577, Japan; 4 Translational Research Institute for Medical Innovation, Osaka Dental University, Osaka 573-1121, Japan; yamamoto-k@cc.osaka-dent.ac.jp; 5 Department of Implementation Science in Public Health, School of Public Health, Kyoto University, Kyoto 606-8501, Japan; takahashi.yoshimitsu.3m@kyoto-u.ac.jp; 6 Center for Innovative Research and Education in Data Science, Institute for Liberal Arts and Sciences, Kyoto University, Kyoto 606-8315, Japan; h_ueshima@kuhp.kyoto-u.ac.jp; 7 Department of Health Informatics, School of Public Health, Kyoto University, Kyoto 606-8501, Japannakayama.takeo.4a@kyoto-u.ac.jp (T.N.); 8 Department of Food Science, Otsuma Women’s University, Tokyo 102-8357, Japan; kiyohara@otsuma.ac.jp

**Keywords:** lung cancer, mass screening, chest X-ray, administrative data

## Abstract

Background: Lung cancer is a major cause of death. Japan has a higher rate of early detection of lung cancer, which is attributed to the impact of chest X-ray examinations implemented as mass screening. This study describes the characteristics and outcomes of patients with lung cancer in Japan, where chest X-ray screening is recommended for everyone aged >40 years old. Methods: This observational study linked the Kyoto City Integrated Database with data from a nationwide cancer registry in Japan. This study assessed individuals aged ≥65 years diagnosed with primary lung cancer between 2014 and 2018. Patients were categorized into the screened or unscreened groups based on their screening history within 1 year before diagnosis. Results: Of 4473 patients with lung cancer, 231 were included in the screened group. The screened group had a mortality rate of 25% at 1.7 years and 50% at 5.6 years, versus 25% at 0.5 years and 50% at 1.8 years for the unscreened group. Conclusions: Patients with primary lung cancer who underwent lung cancer screening had longer survival and better overall health at diagnosis than those who did not undergo screening. Further study is required to estimate the effectiveness of chest X-ray lung cancer screening.

## 1. Introduction

In 2020, an estimated 1.8 million people died of lung cancer, accounting for 18% of all cancer deaths worldwide. It is a leading cause of cancer-related death, with a 5-year survival rate of only 10–20% [1]. At the time of diagnosis, 30–57% of patients have distant metastases [2,3]. To improve the lung cancer survival rate, screenings aimed at early detection and treatment are important.

In Japan, lung cancer has been diagnosed at stage I at approximately 40%, which is higher than that in other countries [3,4], and it has been hypothesized that to be attributable the nation-wide chest X-ray screening system. The nation-wide chest X-ray screening is recommended for citizens aged >40 years by law [5]. Almost all local governments provide screenings for this purpose [6]. Although the effectiveness of chest X-ray as a lung cancer screening tool has not been proven in randomized controlled trials (RCTs) [7,8,9,10], four case–control studies conducted in Japan have suggested a reduction in the odds ratio for lung cancer mortality [11]. However, these case–control studies were all conducted in Japan during the 1990s. Given the decline in smoking rates and improvements in the treatment for lung cancer to date, the effect of these screening methods might have changed, necessitating an understanding of the current situation.

The effectiveness of low-dose computed tomography (LDCT) screening for lung cancer in reducing mortality rates has been demonstrated in meta-analyses [12,13,14,15] and implementation of LDCT is recommended in various countries [16,17,18]. However, it has been tested only among individuals with a smoking history of 20 pack years or more. In addition, the US Preventive Services Task Force reported that Screening high-risk persons with LDCT can reduce lung cancer mortality but also causes false-positive results leading to unnecessary tests and invasive procedures, overdiagnosis, incidental findings, increases in distress, and, rarely, radiation-induced cancers [14]. Based on these concerns, the broad use of LDCT screening in the general population is not recommended. Importantly, 15–20% of men with lung cancer are people who have never smoked, compared with over 50% of women [19]. In Asia, 60–80% of lung cancer cases in women occur in this group [20], and in East Asia, about one third of all lung cancer patients are people who have never smoked [20]. Chest X-ray screening is inexpensive and involves a lower risk of radiation exposure compared with LDCT, and it is implemented as mass screening in Japan. This method could be an appropriate mass screening method for early detection of lung cancer.

We have been conducting studies on the actual situation regarding lung cancer using the Kyoto City Integrated Database (KCIDB), a database integrating medical claims, health checkups, long-term care, and resident registration data [21,22]. In this study, we aim to link the population-based cancer registry implemented nationwide in Japan with the KCIDB to elucidate the actual situation of screening recipients among patients diagnosed with lung cancer and to obtain information that can help reduce the lung cancer mortality rate.

## 2. Materials and Methods

### 2.1. Study Design, Population, and Settings

This observational study was conducted by linking data from the KCIDB and the cancer registry in Japan. Kyoto City is the eighth most populated city in Japan, with approximately 1.5 million people [23].

The details of the KCIDB have been described elsewhere [21,22]. It covers the National Health Insurance, Long-Term Care Insurance, and Advanced Elderly Medical Service Systems; annual lifestyle-related health checkups; and a resident register (deaths and moves into and out of the city). The National Health Insurance system covers self-employed individuals, retirees, and their family members. The Advanced Elderly Medical Service System covers individuals aged ≥75 years and those with disabilities aged 65–74 years. The health-related KCIDB data cover approximately 35% of Kyoto City residents. All KCIDB entries are de-identified and assigned new anonymous personal identifiers by the local government to link data from different systems. This study included data entered between April 2013 and March 2021. The cancer registry was a population-based database managed by local governments until 2015. However, to resolve issues such as duplications when moving between municipalities, a system was established in 2016 whereby the national government aggregates, analyzes, and manages data in a national registry [24]. It registered all cases of cancer diagnosed at a hospital in Japan. For the purpose of linkage with the KCIDB, this study used data from the cancer registry that was registered between January 2013 and December 2020, focusing on lung cancer cases for which the individual’s residential address at the time of diagnosis was within Kyoto City. All data from the cancer registry and KCIDB were anonymized. A unique identification number was assigned to each individual, allowing for the linkage of personal information across the different datasets.

The target population of this study included individuals diagnosed with primary lung cancer for the first time between April 2014 and December 2018. The date of lung cancer diagnosis in the cancer registry data was defined as the index date. In relation to the health insurance scheme mentioned earlier, the population coverage rate for individuals aged 65 and above is relatively high in KCIDB. Additionally, since approximately 70% of lung cancer cases occur in individuals aged 65 and older [2], this study targeted individuals aged 65 and above accordingly. To evaluate primary lung cancer, individuals with a record of lung cancer diagnosis in the cancer registry in 2013 were excluded. In addition, those who were recorded as having moved to Kyoto City after their diagnosis date, those diagnosed less than 180 days after becoming residents, and those aged 75 years at diagnosis who did not have medical claims data in the KCIDB before age 75 were excluded. These exclusions were attributed to a lack of information at the time of diagnosis and uncertainty about whether the lung cancer was primary. Furthermore, those without medical claims data in the KCIDB at the time of diagnosis in the cancer registry were also excluded, as they fell outside the scope of the KCIDB.

This study was reviewed and approved by the ethics committee of the Kyoto University Graduate School and Faculty of Medicine (R3107). Informed consent was waived for this study because it was based on anonymized data and involved no personally identifiable information.

### 2.2. Data Collection and Quality Control

**Figure 1:**
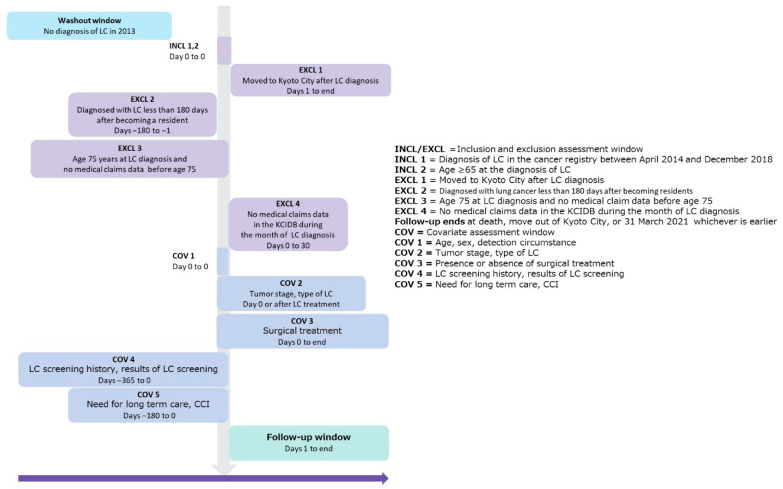
Study design diagram. Abbreviations: LC: lung cancer; KCIDB: Kyoto City Integrated Database; CCI: Charlson Comorbidity Index.

The participants’ sex, age at diagnosis, detection mechanism, screening examination (it includes not only the cancer screening, but also other health checkup), during treatment for other diseases, autopsy, other, or unknown), tumor stage (localized, regional lymph node metastasis, invasion of adjacent structures, distant metastasis, or unknown), type of lung cancer (small cell, non–small cell, or unknown), and existence of hematological treatment (yes, no, or unknown) were extracted. From the KCIDB, comorbidities were identified from the names of diseases listed in the health insurance claim data for the 180 days before the index date using International Classification of Diseases 10th revision (ICD-10) codes. The extraction of disease names related to comorbidities were extracted based on the definition of the Charlson Comorbidity Index (CCI) [25]. The CCI was calculated. Because cancer was included in the evaluation of CCI and every study participant would have at least 1 point, CCI scores were classified as ≤2, 3–4, or ≥5. In addition, disease names with the term “suspicion” were excluded as comorbidities. Information on the need for long-term care or support as part of the Long-Term Care Insurance System was collected on the basis of the most recent certification made within 6 months before the index date. The Long-Term Care Insurance System categorizes levels of care services on the basis of the need for assistance with activities of daily living as 25 to <32 min per day (Level 1 need for support), 32 to <50 min per day (Level 2 need for support or Level 1 need for care), 50 to <70 min per day (Level 2 need for care), 70 to <90 min per day (Level 3 need for care), 90 to <110 min per day (Level 4 need for care), or ≥110 min per day (Level 5 need for care) [26,27]. Survival, death, or relocation was confirmed on the basis of the resident registry included in the KCIDB. The observation period was set to last until March 31, 2021. As the information in the resident register was in units of years and months, survival was calculated in months (Figure 1).

### 2.3. Statistical Analysis

Patients were categorized into two groups based on their lung cancer screening history: those who had received lung cancer screening within 1 year before the diagnosis of lung cancer and those who had not. To visualize differences between groups, data were summarized in a table. Patient background information included age, sex, lung cancer screening results, detection circumstance, need for long-term care, type of lung cancer, and CCI. Information regarding outcomes included disease stage at diagnosis and surgical treatment. Kaplan–Meier analysis was used to plot survival curves. For continuous variables, mean and standard deviation (SD) were reported. For categorical scales, proportions were presented. As indicators of group differences, *p*-values were obtained from *t*-tests for continuous variables and chi-square tests or Fisher’s exact tests for categorical variables.

Descriptive statistics was performed using SQL Server Management Studio, version 15.0 (Microsoft Corporation). Survival curves were plotted using Stata/MP, version 17 (StataCorp). *t*-tests, chi-square tests, and Fisher’s exact tests were performed using a two-sided significance level of 0.05, and all analyses were conducted with R version 4.3.2.

## 3. Results

**Figure 2:**
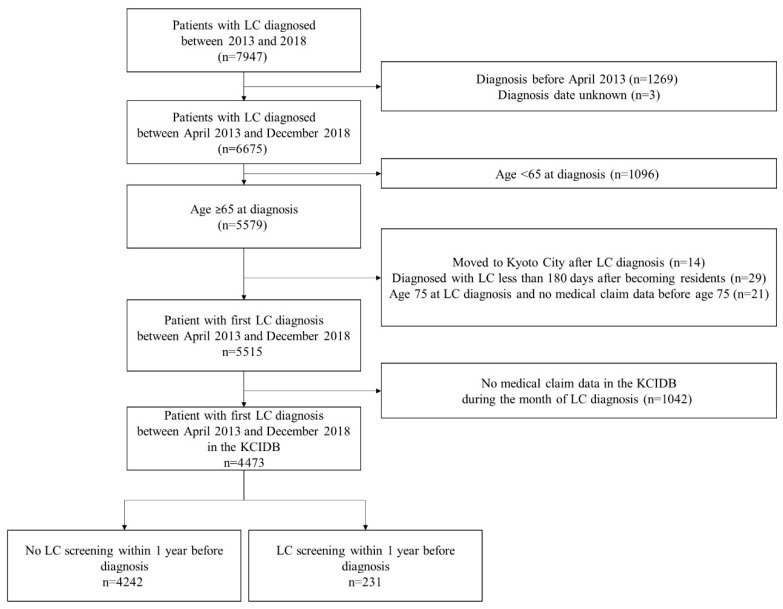
Patient flow. Abbreviations: LC: lung cancer; KCIDB: Kyoto City Integrated Database.

During the study period, 7947 patients were registered as being diagnosed with lung cancer in the cancer registry. Patients with unknown diagnosis dates and those diagnosed before March 2013 were excluded, leaving 6675 patients diagnosed on or after 1 April 2014. We excluded patients under the age of 65 at diagnosis, those who could not be assumed to have incident lung cancer, and those without medical claims data from the month of lung cancer diagnosis, leaving 4473 individuals who were included in the study (Figure 2). Of the 4473 individuals, 231 (5.2%) had undergone lung cancer screening within 1 year before diagnosis, whereas the remaining 4242 had no history of lung cancer screening within 1 year before diagnosis.

Table 1 presents the background characteristics of each group. There were 135 males (58.4%) in the screened group and 2696 males (63.6%) in the unscreened group. The average age was 75.4 (SD 6.1) years in the screened group and 77.8 (SD 7.1) years in the unscreened group. In the screened group, 96 individuals (41.6%) were determined to have required a detailed examination due to suspected lung cancer and 45 individuals (19.5%) were determined to have required a detailed examination due to suspicions of conditions other than lung cancer. The detection circumstances recorded in the cancer registry showed that the most common circumstance in the screened group was detection by screening examination (*n* = 103, 44.6%), followed by incidental detection during treatment for other diseases (*n* = 78, 33.8%), other (*n* = 26, 11.3%), and unknown (*n* = 24, 10.4%). No cases were detected through autopsy. In the unscreened group, incidental detection during treatment for other diseases was the most common (*n* = 2139, 50.4%), followed by other (*n* = 1103, 26.0%), unknown (*n* = 766, 18.1%), detection during screening (*n* = 231, 5.4%), and autopsy (*n* = 3, 0.1%). Regarding the type of lung cancer, non–small cell lung cancer was the most common in both groups, followed by unspecified and small cell lung cancer. In the screened group, 83.1% of participants had non–small cell lung cancer, whereas 66.8% of participants in the unscreened group had non–small cell lung cancer. The proportion of unspecified cancer types was 13.4% (*n* = 24) in the screened group and 25.7% (*n* = 1092) in the unscreened group. The proportion of patients who needed long-term care at diagnosis was 5.2% (*n* = 12) in the screened group and 22.5% (*n* = 955) in the unscreened group. In the screened group, 34.2% had a score of ≥5, 39.0% had a score of 3–4, and 26.8% had a score of ≤2. In the unscreened group, 57.6% had a score of ≥5, 33.6% had a score of 3–4, and 8.8% had a score of ≤2.

Table 2 shows the outcomes for both groups. At the time of lung cancer diagnosis, 51.9% of the screened group were diagnosed with localized disease, the most common category. By contrast, the unscreened group had the highest proportion of distant metastasis detections, 36.6%. In addition, 49.8% of the screened group and 30.4% of the unscreened group underwent surgical treatment.

**Figure 3:**
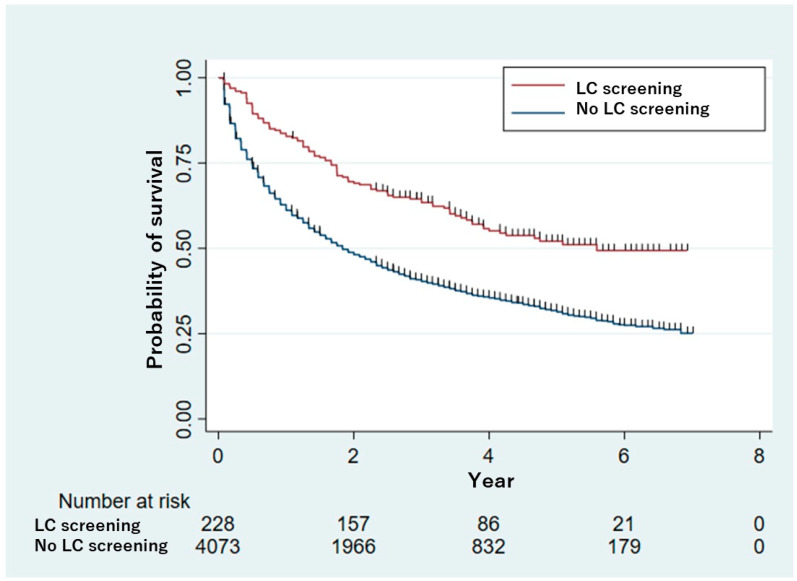
Kaplan–Meier survival curves by initial treatment for NSCLC. Abbreviation: NSCLC, non–small cell lung cancer.

Figure 3 presents the survival curves for both groups. The mortality rate in the screened group was 25% at 1.7 years and 50% at 5.6 years. However, in the unscreened group, the mortality rate was 25% at 0.5 years and 50% at 1.8 years.

## 4. Discussion

This study linked a database that integrates medical, health, medical checkup, nursing care, and residential data covering much of the elderly population in Kyoto City with a population-based cancer registry that has been implemented throughout Japan. The background characteristics and outcomes of patients with initial lung cancer were described according to lung cancer screening status.

Japan, with its world-renowned universal health insurance system, provides comprehensive medical care and performs large-scale cancer screenings by law. Moreover, as an area that is experiencing super-aging ahead of the rest of the world, the results of this study, which covers a large proportion of the elderly population, might provide insights into lung cancer medical strategies in aging societies that are anticipated to emerge in various countries in the future.

Patients with lung cancer who have undergone lung cancer screening had longer survival than those who did not undergo lung cancer screening. Even in a large-scale RCT that verified the effectiveness of chest X-ray, it has been reported that lung cancer detected by chest X-ray is of low stage [28]. Findings from the present study suggest that early detection through screening, which resulted in less severe disease at detection and a higher proportion of curative treatments at the time of diagnosis, could lead to more surgical treatment and potentially better outcomes. However, it is imperative to acknowledge that individuals participating in screening present with a higher baseline health status at the time of diagnosis compared to nonparticipants, characterized by lower care requirements and CCI scores. Furthermore, this study was not able to exclude lead-time bias and length biases [29]. Lead-time bias refers to an apparent extension in survival due to earlier disease diagnosis through screening. The length bias reflects an apparent extension in survival due to the slow progression of cancers detected by screening. In addition, not all patients, especially those with higher care needs or higher CCI scores, can undergo screening, indicating that lung cancer screening might not be universally applicable.

In this study, among 4473 elderly individuals newly diagnosed with lung cancer between 2014 and 2018, only 231 (5.4%) had undergone lung cancer screening within one year prior to diagnosis. This indicates that, at present, the effect of lung cancer screening among patients with lung cancer remains limited. In 2022, the lung cancer screening participation rate among individuals aged 40–69 was 49.7% nationwide in Japan, whereas in Kyoto City it was 39.2%, representing a lower level [30]. To enhance the impact of lung cancer screening on patients, improving participation rates is essential.

In this study, incidental detection during treatment for other diseases was the most common detection pathway in unscreened patients. Prior studies have indicated that incidental detection of lung cancer can contribute with improved outcomes compared to detection through the evaluation of symptoms [28]. According to a 2020 patient survey, approximately 69% of citizens aged ≥65 years visit outpatient clinics [31]. However, regular chest X-ray screenings are typically performed only in patients visiting cardiology or pulmonology departments. Therefore, extending these screenings to consultations in other medical departments or recommending cancer screenings to patients may potentially improve outcomes.

As an alternative large-scale screening method among people who have never smoked and those who smoke lightly, LDCT is also being investigated. In Japan, a large-scale RCT is currently underway to evaluate the effectiveness of LDCT among this group, which is scheduled to be completed in 2035 [32]. At present, an observational study conducted in Hitachi City, Japan, provides evidence regarding LDCT among them. The study reported that the 5-year survival rate of patients with lung cancer detected through health checkups including LDCT was 90% [33], and that the standardized mortality ratio for lung cancer, including among people who have never smoked and those who smoke lightly, significantly decreased at both 4 and 8 years after the introduction of LDCT screening [34]. On the other hand, in 2025, the National Cancer Center issued lung cancer screening guidelines stating that, aside from people who smoke heavily (defined as those with a smoking index [number of cigarettes per day × years] of 600 or more), evidence for LDCT screening remains insufficient, and therefore its implementation is not recommended [18]. Taking together with the results of the present study, no conclusion has yet been reached regarding the optimal screening method for people who have never smoked and those who smoke lightly, and further accumulation of broad evidence is required.

This study had several limitations. The study participants were patients registered in a population-based cancer registry and were limited to those whose data could be linked with the KCIDB to obtain patient details. Due to the limitations of the database, this study focused on individuals aged 65 years and older. Approximately 30% of the lung cancer participants were excluded from this study. Currently, chest X-ray screening is recommended in Japan for individuals aged 40 and above, and a separate investigation is needed for those aged 40–64. Lung cancer screening could have occurred through other mechanisms, such as employer-sponsored examinations or during comprehensive health checkups that individuals can choose to attend. Those who underwent such screenings were classified as nonrecipients of lung cancer screening in this study, which could underestimate the effects of lung cancer screening. This study did not include those who received lung cancer screening but did not have lung cancer. Finally, as mentioned earlier, assessing the effectiveness of lung cancer screening in this study is inappropriate. Thus, it does not verify the effect of lung cancer screening itself. Future studies are needed to compare the outcomes between those who have and have not undergone lung cancer screening among the target population for lung cancer screening and to verify the effects of lung cancer screening.

## 5. Conclusions

In patients with primary lung cancer, those who underwent lung cancer screening had longer survival and better overall health at diagnosis compared with those who did not undergo screening. However, further studies are required to estimate the effectiveness of chest X-ray lung cancer screening.

## Figures and Tables

**Table 1:** Backgrounds of patient with LC according to LC screening within one year before the diagnosis.

	With LC Screening Within One Year Before the Diagnosis	Without LC Screening Within One Year Before the Diagnosis	*p*
	*n* = 231	*n* = 4242	
Male, *n* (%)	135 (58.4)	2696 (63.6)	0.13
Age mean (SD)	75.4 (6.1)	77.8 (7.1)	<0.001
Result of LC screening			
Requires a detailed examination (not suspicion for LC)	45 (19.5)	NA	
Requires a detailed examination (suspicion for LC)	96 (41.6)	NA	
Detection circumstances, *n* (%)			
Screening examination	103 (44.6)	231 (5.4)	<0.001
Incidental detection in treatment of other diseases	78 (33.8)	2139 (50.4)	
Autopsy	0 (0.0)	3 (0.1)	
Others	26 (11.3)	1103 (26.0)	
Unknown	24 (10.4)	766 (18.1)	
Type of LC, *n* (%)			
Small cell	8 (3.5)	317 (7.5)	<0.001
Non-small cell	192 (83.1)	2833 (66.8)	
Unspecified	31 (13.4)	1092 (25.7)	
Need for long-term care, *n* (%)	12 (5.2)	955 (22.5)	<0.001
CCI			
≤2	62 (26.8)	373 (8.8)	<0.001
3~4	90 (39.0)	1427 (33.6)	
5≤	79 (34.2)	2442 (57.6)	

LC indicates Lung Cancer, SD: standard deviation, CCI: Charlson comorbidity index.

**Table 2:** Outcomes.

		With LC Screening Within One Year Before the Diagnosis	Without LC Screening Within One Year Before the Diagnosis	*p*
		*n* = 231	*n* = 4242	
Tumor stage, *n* (%)	Intraepithelial	2(0.9)	29(0.7)	<0.001
	Localized	120(51.9)	1486(35.0)	
	Regional lymph node metastasis	19(8.2)	415(9.8)	
	Invasion to adjacent structures	17(7.4)	335(7.9)	
	Distant metastasis	58(25.1)	1552(36.6)	
	Unknown	15(6.5)	422(9.9)	
	Missing data	0(0.0)	3(0.1)	
Surgical treatment, *n* (%)	Yes	115(49.8)	1287(30.3)	<0.001
	No	114(49.4)	2876(67.8)	
	Unknown	2(0.9)	68(1.6)	
	Missing data	0(0.0)	11(0.3)	

LC indicates Lung Cancer.

## Data Availability

The datasets presented in this article are not readily available because the data was used under contract for the current study. Data are not publicly available.
